# p53R172H and p53R245W Hotspot Mutations Drive Distinct Transcriptomes in Mouse Mammary Tumors Through a Convergent Transcriptional Mediator

**DOI:** 10.1158/2767-9764.CRC-24-0128

**Published:** 2024-08-09

**Authors:** Joy M. McDaniel, Rhiannon L. Morrissey, Denada Dibra, Lalit R. Patel, Shunbin Xiong, Yun Zhang, Gilda P. Chau, Xiaoping Su, Yuan Qi, Adel K. El-Naggar, Guillermina Lozano

**Affiliations:** 1 Department of Genetics, The University of Texas MD Anderson Cancer Center, Houston, Texas.; 2 Genetics and Epigenetics Graduate Program, The University of Texas MD Anderson Cancer Center UTHealth Houston Graduate School of Biomedical Sciences, Houston, Texas.; 3 Department of Pharmaceutical Sciences, College of Pharmacy and Health Sciences, Texas Southern University, Houston, Texas.; 4 Department of Bioinformatics and Computational Biology, The University of Texas MD Anderson Cancer Center, Houston, Texas.; 5 Department of Pathology, The University of Texas MD Anderson Cancer Center, Houston, Texas.

## Abstract

Aggressive breast cancers harbor *TP53* missense mutations. Tumor cells with *TP53* missense mutations exhibit enhanced growth and survival through transcriptional rewiring. To delineate how *TP53* mutations in breast cancer contribute to tumorigenesis and progression *in vivo*, we created a somatic mouse model driven by mammary epithelial cell-specific expression of *Trp53* mutations. Mice developed primary mammary tumors reflecting the human molecular subtypes of luminal A, luminal B, HER2-enriched, and triple-negative breast cancer with metastases. Transcriptomic analyses comparing *MaP*^*R172H/−*^ or *MaP*^*R245W/−*^ mammary tumors to *MaP*^*−/−*^ tumors revealed (1) differences in cancer-associated pathways activated in both p53 mutants and (2) Nr5a2 as a novel transcriptional mediator of distinct pathways in p53 mutants. Meta-analyses of human breast tumors corroborated these results. *In vitro* assays demonstrate mutant p53 upregulates specific target genes that are enriched for Nr5a2 response elements in their promoters. Co-immunoprecipitation studies revealed p53R172H and p53R245W interact with Nr5a2. These findings implicate NR5A2 as a novel mediator of mutant p53 transcriptional activity in breast cancer.

**Significance:**

Our findings implicate NR5A2 as a novel mediator of mutant p53 transcriptional activity in breast cancer. NR5A2 may be an important therapeutic target in hard-to-treat breast cancers such as endocrine-resistant tumors and metastatic triple-negative breast cancers harboring *TP53* missense mutations.

## Introduction

Breast cancer comprises four subtypes: luminal A, luminal B, HER2-enriched, and basal-like breast cancer (1, 2). Luminal breast cancers are driven by estrogen receptor (ER) and/or progesterone receptor (PR). HER2-enriched breast cancers are driven by amplification and constitutive signaling of HER2. Basal-like breast cancers are often termed triple-negative breast cancer (TNBC) owing to the lack of expression of ER, PR, and HER2. TNBCs are often metastatic and chemoresistant. Development of anti-hormonal and anti-HER2 therapies has revolutionized breast cancer treatment (1–8). However, there is a need to develop targeted therapies for aggressive breast cancers.

A molecular driver associated with aggressive breast cancer is the mutation of *TP53*. Recent studies show that *TP53* mutations are the most frequent alteration in metastatic breast cancers (9, 10). *TP53* mutations are associated with endocrine resistance in luminal breast cancers, targeted therapy resistance in HER2-enriched breast tumors; and chemoresistance in TNBCs (11–14). Overall, *TP53* mutations are present in 34% of all breast cancers: luminal A (12%); luminal B (29%), HER2-enriched (72%); and TNBC (88%; ref. 15). Therefore, mutations in *TP53* are early events in mammary tumorigenesis that likely play a role in both initiation and progression of aggressive breast tumors.

The *TP53* tumor suppressor gene encodes a transcription factor that functions as a central sensor of cell signals and is a master regulator of cell response to DNA damage (16, 17). The most common *TP53* alterations are missense mutations in the DNA-binding domain (16, 18). Modification of any five arginine residues that are major hotspots for p53 mutations in human cancers disables p53 sequence-specific DNA binding (19). In addition to causing loss of wild-type (WT) tumor suppressor activity, some p53 missense mutations also confer growth and survival advantages including enhanced transformation, greater invasion, increased metastatic potential, and chemoresistance that surpasses what is observed in cells lacking wild-type p53 (20–25). Mutant p53 activities are often mediated through interactions with other DNA-binding proteins to activate the transcription of genes (26, 27), many of which play roles in tumor development. In breast cancer, mutant p53 interacts with SREBP2 to activate the cholesterol biosynthesis pathway driving the disorganized cell morphology of spheroids (21). A functional p53 N-terminal activation domain is required for oncogenic activities of mutant p53 (21, 28, 29).

We developed a physiologically relevant somatic breast cancer mouse model, in which *Trp53*^*R172H*^ and *Trp53*^*R245W*^ missense mutations (orthologs to the human breast cancer hotspot missense mutations *TP53*^*R175H*^ and *TP53*^*R248W*^), are focally induced (25). *Trp53*^*R172H/−*^, *Trp53*^*R245W/−*^, and *Trp53*^*−/−*^ mice were generated *via* mammary gland injection of adenovirus containing Cre-recombinase (Ad-Cre). Recombination in the mammary gland results in excision of WT *Trp53* complementary DNA (cDNA) sequences from the endogenous locus, resulting in mammary ductal epithelial cell-specific mutant p53 expression of *Trp53*^*R172H*^ or *Trp53*^*R245W*^ and biallelic loss of *Trp53* (referred to as *MaP*^*R172H*^, *MaP*^*R245W*^, or *MaP*^*−*^ from hereon). Mice with these mutant *Trp53* alleles develop breast tumors that spontaneously disseminate and form metastases (25).

Transcriptomic analyses comparing tumors from *MaP*^*R172H/−*^ and *MaP*^*R245W/−*^ mice to those of *MaP*^*−/−*^ mice revealed (i) dysregulation of unique cancer-associated pathways in tumors and (ii) Nr5a2 (nuclear receptor subfamily 5 group A member 2) as a predicted mediator of mutant-p53 transcriptional programs.

NR5A2 is an orphan nuclear hormone receptor that belongs to the NR5A (Ftz-F1) subfamily of nuclear receptors and is predominantly expressed in the enterohepatic axis and ovary (30, 31). Studies support a role in embryonic stem cell differentiation and development of several cancers (32–38). Although NR5A2 has been associated with poor prognosis in breast cancer (39, 40), its role in breast cancer is not fully understood. Our findings suggest NR5A2 is a novel mediator of mutant p53 transcriptional rewiring in breast cancer.

## Materials and Methods

### Mice

Female *MaP*^*R172H/−*^, *MaP*^*R245W/−*^, and *MaP*^*−/−*^ mice in an F1 hybrid 50% BALB/c and 50% C57BL6/J background were generated *via* mammary gland injection of adenovirus containing Cre-recombinase (Ad-Cre) at the age of 10 to 12 weeks, as previously described (25). Genotyping analysis of all mice was performed by PCR as previously described (25). Mouse cohorts were monitored daily for tumor development. Moribund mice were euthanized according to Institutional Animal Care and Use Committee guidelines, and tissues were collected in 10% v/v formalin and paraffin-embedded. Additionally, a portion of mammary tumors and matching metastases were flash-frozen on dry ice and stored for downstream analyses. All mouse experiments were performed in compliance with the guidelines of the Association for Assessment and Accreditation of Laboratory Animal Care and the US Public Health Service Policy on Human Care and Use of Laboratory Animals. All animal studies and procedures were approved by the Institutional Animal Care and Use Committee at MD Anderson Cancer Center.

### Molecular subtyping

Molecular subtyping of mammary tumors to define the expression of *Esr1* (ER), *Pgr* (PR), and *Erbb2* (HER2) was performed *via* qRT-PCR analysis as previously described (25).

## Histology

Mammary tissues harvested from mice were fixed in 10% neutral buffered formalin, followed by paraffin embedding; 4-μm tissue sections were stained with hematoxylin and eosin by the MD Anderson Cancer Center Department of Veterinary Medicine Surgery and Histology Laboratory. Tissue sections were analyzed by a pathologist. Hematoxylin and eosin bright field images were taken at 40X using the Nikon Eclipse Ni microscope.

### Immunofluorescence staining

Paraffin-embedded tumor sections were deparaffinized followed by rehydration. Tris(hydroxymethyl) aminomethane–ethylenediaminetetraacetic acid pH 9.0 was used for antigen retrieval. PBS containing 3% fish gelatin (VWR) was used to block slides for 20 minutes. To visualize the TdTomato reporter, immunofluorescence staining of TdTomato was performed on unstained tissue sections using a rabbit polyclonal RFP antibody (Cat # 600-401-379, Rockland Immunochemicals, 1:200). Tissue sections were counterstained with DAPI (Thermo Fisher Scientific). Nikon Eclipse 80i Advanced Research Microscope (Nikon, RRID:SCR_015572) and NIS-Elements imaging software (Nikon, RRID:SCR_014329) were used to acquire images.

### RNA extraction

A portion of the mouse mammary tumor was homogenized using TRIzol and the Qiagen RNeasy Mini Kit (Qiagen Cat # 74104, RRID:SCR_008539) to isolate total RNA using a modified extraction protocol. TRIzol was added to tissue homogenates and incubated at room temperature for 5 minutes. A 1:5 volume of chloroform was added to the tissue/TRIzol mixture and vortexed briefly. Samples were incubated at room temperature for 3 minutes, followed by centrifugation at 12,000 *g* at 4°C for 30 minutes. The aqueous phase was transferred to a new collection tube before mixing with 1.5 volume of 100% ethanol and loading onto an RNeasy spin column (Qiagen, CA). Columns were centrifuged at >8,000 *g* for 15 seconds. Flow-through for each sample was discarded. Each column was washed with buffer RW1, treated with DNase I, and then washed with buffers RW1 and RPE, respectively. Residual ethanol was dried with a final spin and RNA was eluted in 60 μL of nuclease-free water.

Total RNA was isolated from cultured cell lines either overexpressing p53R172H or p53R245W or harboring *Nr5a2* knockdown using TRIzol reagent and the Zymo Research Direct-zol RNA Microprep kit (Zymo Research Cat #R2060, RRID:SCR_008968), following the manufacturer’s instructions.

### RNA sequencing

Libraries were generated and sequenced by the MD Anderson Cancer Center Advanced Technology Genomics Core on an Illumina HiSeq 4000 (*MaP*^*R172H/−*^ tumors; RRID:SCR_016386) or Illumina NovaSeq 6000 Sequencing System (RRID:SCR_016387; *MaP*^*R245W/−*^ and *MaP*^*−/−*^ tumors). Barcoded Illumina-compatible stranded total RNA libraries were prepared using the TruSeq Stranded Total RNA Sample Preparation Kit (Illumina). Briefly, 197 to 250 ng of DNase I treated total RNA was depleted of cytoplasmic and mitochondrial ribosomal RNA using Ribo-Zero Gold (Illumina). After purification, the RNA was fragmented using divalent cations, and double-stranded cDNA was synthesized using random primers. The ends of the resulting double-stranded cDNA fragments were repaired, 5′-phosphorylated, 3′-A tailed, and Illumina-specific indexed adapters were ligated. The products were purified and enriched with 12 cycles of PCR to create the final cDNA library. The libraries were quantified using the Qubit dsDNA HS Assay Kit and assessed for size distribution using the Agilent TapeStation System (RRID:SCR_018435; Agilent Technologies). The libraries for *MaP*^*R127H/−*^ tumors were then pooled, 7 to 8 libraries per pool for a total of three pools. The libraries corresponding to *MaP*^*R245W/−*^ and *MaP*^*−/−*^ tumors were then pooled, with 18 samples per pool. All library pools were quantified by qPCR using the KAPA Library Quantification Kit (Kapa Biosystems). The library pools for *MaP*^*R172H/−*^ tumors were sequenced, one pool per lane on the Illumina HiSeq 4000 sequencer using the 76-nt paired-end format. The library pool for *MaP*^*R245W/−*^ and *MaP*^*−/−*^ tumors was sequenced on the NovaSeq 6000 SP-200 flow cell using the 100-nt paired-end format.

### Transcriptome analysis of mouse mammary tumors

FASTQ files were analyzed for read and base quality using FastQC (RRID:SCR_014583, v 0.11.9). Files that passed QC were analyzed for gene expression. Briefly, reads were aligned using STAR (RRID:SCR_004463, v 2.7.10b) with default parameters to the mm39 (GRCm39) reference genome (41) and gene expression was determined from mapped reads counted using RSEM (RRID:SCR_000262, v 1.3.3; ref. 42) using the GENCODE (RRID:SCR_014966, v M34) annotation of *Mus musculus* genes (43). Raw counts were normalized and compared for differential expression using *DESeq2* (RRID:SCR_000154; ref. 44). To account for multiple comparisons, *P* values were adjusted to FDRs using the Benjamini–Hochberg method (45). Differentially expressed genes (DEG) were identified using statistical cutoffs of log fold change of ≥1.0 or ≤−1.0 and FDR ≤5%. DEGs were assessed for pathway enrichment using gene set enrichment analysis (GSEA; RRID:SCR_003199) and MSigDB from the Molecular Signature Database (RRID:SCR_016863, v 2023.2.Hs; ref. 46).

### Microarray data analysis

The METABRIC study was queried for human breast tumor samples harboring a *p53*^*R175H*^ or *p53*^*R248Q/W*^ mutation in cBioPortal (47, 48). This study was also queried for human breast tumors with biallelic deletion of *TP53*. Sample IDs for each tissue were used to acquire corresponding microarray data. Gene expression microarray results matching the identified samples were processed using the Bioconductor package *limma* for differential expression (RRID:SCR_006442; ref. 49). GSEA was performed using Hallmark pathways from the Molecular Signature Database and DEGs between samples with *TP53* missense mutations and samples with homozygous *TP53* deletions.

### Transcription factor motif discovery analysis

FASTA files containing sequences from the 10,000 base pairs upstream of the transcriptional start site of significantly upregulated genes were downloaded using the Ensembl Biomart (RRID:SCR_002344; ref. 50) and used as input for MEME or MEME-SEA (MEME-Suite, Motif-based sequence analysis tools, RRID:SCR_001783) to identify significantly enriched motifs (bioRxiv 2021.08.23.457422; refs. 51,52). In a reciprocal analysis, FIMO (MEME-Suite, Motif-based sequence analysis tools, RRID:SCR_001783) was used to identify genes with promotors that harbor at least one motif corresponding to a defined transcription factor’s known DNA-binding sequence (53).

### Overexpression of murine p53 mutants

Lentivirus expressing murine p53R172H- or p53R245W-mutant proteins (and enhanced green fluorescent protein) was synthesized by Vector-Builder. A cell line was derived from a *Trp53*-null mammary tumor using previously described methods (54). The cell line was transduced with lentivirus, selected with 4-μg puromycin for 2 days, and assessed for transduction by enhanced green fluorescent protein expression on a fluorescence microscope. Transduced cells were washed with PBS and collected for protein or RNA extraction. The in-house generated cell line used for transduction experiments was authenticated for the *Trp53-*null genotype and for murine origin. Cells were cultured for 10 passages between thawing, transduction, and RNA extraction.

### Nr5a2 knockdown

*MaP* ^*R172H/−*^ and *MaP*^*R245W/−*^ mammary tumor-derived cell lines were transfected with either *Nr5a2* siRNAs (si-1, si-2, si-3, and si-4) or universal nontargeting control siRNA (J-047044-09-0010, J-047044-10-0010, J-047044-11-0010, J-047044-12-0010, and D-001810-10-20, respectively, Horizon Discovery). Total RNA was isolated 48 hours after the transfection as described above. Mammary tumor-derived cell lines used for *Nr5a2* knockdown experiments were authenticated for the *Trp53*^*R172H/−*^ or *Trp53*^*R245W/−*^ genotype and for murine origin. Cells were cultured for 10 passages between thawing, transduction, and RNA extraction.

### Protein isolation, immunoprecipitation, and immunoblotting

Protein was isolated from cell pellets using NP-40 lysis buffer containing a protein inhibitor cocktail (Sigma-Aldrich). Supernatant was collected and protein was quantified utilizing the Pierce BCA Protein Assay Kit (Thermo Fisher). The supernatant was mixed with 6× SDS-PAGE gel loading buffer. Samples were boiled for 5 minutes at 95°C to denature protein. Protein lysates were resolved on 10% SDS-PAGE gels and immunoblotted with antibodies against p53 (CM5, P53-CM5P-L; Leica Biosystems; 1:1,000), Nr5a2, (Anti-NR5A2 (LRH1), ABE2867; EMD-Millipore; 1:1,000), anti-rabbit IgG peroxidase-conjugated [Goat anti-Rabbit IgG (H + L) Cross Adsorbed Secondary Antibody, HRP, A16104, Invitrogen; 1:6,000], anti-mouse IgG peroxidase-conjugated [Goat anti-mouse IgG (H + L) Secondary Antibody, HRP, 62 to 6520, Invitrogen; 1:6,000] and vinculin [Anti-Vinculin (E1E9V), #13901; Cell Signaling; 1:500].

For immunoprecipitation, tumor tissue pieces were homogenized followed by lysis with a nondenaturing buffer [50-mmol/L tris HCl (pH 7.4), 150-mmol/L NaCl, 1% NP-40, and 2-mmol/L ethylenediaminetetraacetic acid containing protease and phosphatase inhibitors (Santa Cruz Biotechnology)]. Immunoprecipitations containing 4 μL of primary rabbit antibody against NR5A2 [Anti-NR5A2 (LRH1), ABE2867; EMD-Millipore] or rabbit IgG (Cell Signaling Technology) and 1 mg of total protein lysate were performed overnight at 4°C. The following day, lysates were incubated with a 20-μL of Protein A Dynabeads (Dynabeads Protein A for Immunoprecipitation, 10001D, Thermo Fisher Scientific) for 3 hours at 4°C. The Dynabeads were washed four times and heated to 95°C for 5 minutes in 35 μL of sample buffer. For the immunoblotting step, equal amounts of protein from each sample were subjected to 10% SDS-PAGE followed by transfer to Immun-Blot polyvinylidene difluoride membranes (Bio-Rad). The following antibodies were used to probe the blot: anti-p53 (CM5, P53-CM5P-L; Leica Biosystems), anti-NR5A2 [Anti-NR5A2 (LRH1), ABE2867; EMD-Millipore], anti-vinculin [Anti-Vinculin (E1E9V), #13901; Cell Signaling], anti-rabbit IgG peroxidase-conjugated [Goat anti-Rabbit IgG (H + L) Cross Adsorbed Secondary Antibody, HRP, A16104, Invitrogen; 1:6,000], and VeriBlot-horseradish peroxidase (VeriBlot-HRP, ab131366, Abcam).

### Real-time qRT-PCR of p53 mutant-specific target genes

Total RNA isolated from cell lines overexpressing p53R172H and p53R245W or transfected with control and *Nr5a2* siRNAs was used to prepare cDNA *via* reverse transcription using the iScript cDNA synthesis kit (Bio-Rad). qRT-PCR was performed using SYBR green (Bimake) on the Bio-Rad CFX384 Real-Time Detection System (RRID:SCR_018057; Bio-Rad). Relative expression levels for murine p53 mutant-specific target genes of interest or *Nr5a2* were normalized to *Actab*. A description of the primers used is in Supplementary Table S1.

### Statistical analysis

Kaplan–Meier survival analyses for each mouse cohort were performed using Prism 9 software (GraphPad Software, v 9, RRID:SCR_002798, CA). Fisher’s exact tests comparing primary tumor incidence were performed using Prism 9 software. Mann–Whitney *U* tests on real-time qPCR values were performed using Prism 9 software. Statistical analysis of changes in gene expression was performed using the Bioconductor packages *limma* and *DESeq2* in the R Project for Statistical Computing environment (RRID:SCR_001905, v 4.2.0).

### Data availability

RNA-seq data reported in this article have been deposited in the Gene Expression Omnibus database, GSE270515.

## Results

### Somatic breast cancer mouse models driven by p53 mutations recapitulate human tumor diversity

**Figure 1:**
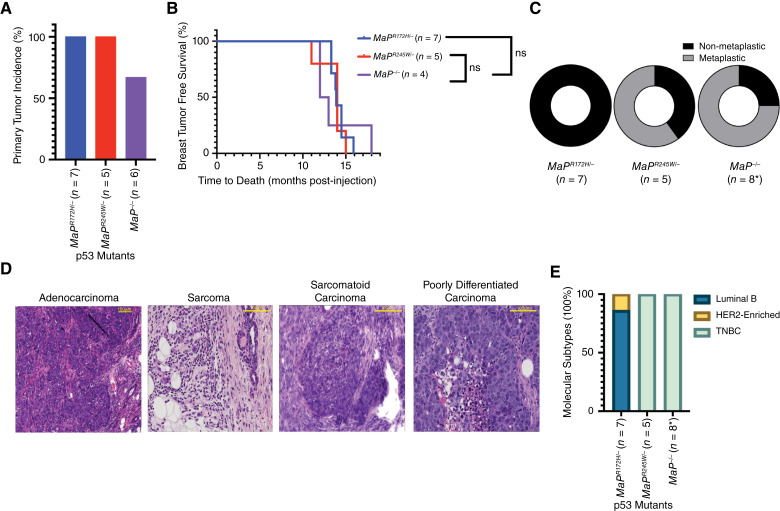
Molecular characterization of mammary tumors from *MaP* mice. **A,** Incidence of primary mammary tumor formation across *MaP*^*R172H/−*^ (*n* = 7), *MaP*^*R245W/−*^ (*n* = 5), and *MaP*^*−/−*^ (*n* = 6) mice. **B,** Tumor-free survival of mammary tumor-bearing mice. **C,** Histological subtypes across *MaP* mice (*Indicates four mice had two primary tumors). **D,** Histology of mammary tumors in *MaP* mice. Yellow scale bar, 100 µm. **E,** Breast cancer molecular subtypes defined by qPCR for *Esr1* (Estrogen Receptor), *Pgr* (progesterone receptor), and *Erbb2* (HER2).

To examine how p53R172H and p53R245W drive mammary tumor development and progression, we generated and examined mice with the following mutations: *MaP*^*R172H/−*^ (*n* = 7), *MaP*^*R245W/−*^ (*n* = 5), and *MaP*^*−/−*^ (*n* = 6). Adenovirus containing Cre-recombinase (Ad-Cre) was delivered to the mammary epithelial duct *via* intraductal injection. Ad-Cre allows recombination of the WT *Trp53* allele to express mutant p53. Thus, mutant p53 is only expressed in the epithelial cells, while the stroma and immune components retain WT *Trp53* (25). *MaP*^*R172H/−*^ mice resulted in 100% mammary tumor formation, with a median latency of 13.9 months postinjection (Table 1; Fig. 1A and B). Moreover, 100% of *MaP*^*R245W/−*^ mice developed primary mammary tumors with a median latency of 14 months postinjection (Table 1; Fig. 1A and B). Only 67% (4/6) of Ad-Cre injected *MaP*^*-^/^-*^ mice developed mammary tumors, with a median latency of 14.7 months postinjection (Table 1; Fig. 1A and B). No statistically significant differences in tumor-free survival were observed (Fig. 1B).

**Table 1:** Tumors Used in Study

Mouse ID	Genotype	Histological classification	Molecular subtype	Metastasis	Latency (months postinjection)
*Ma* ^*PR172H/−*^					
YZ2^a^	*MaP* ^*R172H/−*^; *Rosa26*^*LSL-TdTomato/+*^	Nonmetaplastic AC	Luminal B	Lung	13.8
YZ3^a^	*MaP* ^*R172H/−*^	Nonmetaplastic AC	Luminal B	No	13.3
YZ4^a^	*MaP* ^*R172H/−*^	Nonmetaplastic AC	Luminal B	No	14.5
YZ6^a^	*MaP* ^*R172H/−*^; *Rosa26*^*LSL-TdTomato/+*^	Nonmetaplastic AC	HER2-enriched	No	15.9
YZ7^a^	*MaP* ^*R172H/−*^	Nonmetaplastic AC	Luminal B	No	13.3
YZ10^a^	*MaP* ^*R172H*/*−*^; *Rosa26*^*LSL−TdTomato/+*^	Nonmetaplastic AC	Luminal B	Lung	13.9
YZ11^a^	*MaP* ^*R172H/−*^	Nonmetaplastic AC	Luminal B	No	14.5
*MaP* ^*R245W/−*^					
JM1126^a^	*MaP* ^*R245W/−*^	Nonmetaplastic SAC	TNBC	Lung	14
JM1133^a^	*MaP* ^*R245W/−*^	Metaplastic SC	TNBC	Lung	14
JM1233^a^	*MaP* ^*R245W/−*^; *Rosa26*^*LSL-TdTomato/+*^	Metaplastic SC	TNBC	Lung	14
JM1564^a^	*MaP* ^*R245W/−*^; *Rosa26*^*LSL-TdTomato/+*^	Nonmetaplastic AC	TNBC	Lung	15
RM0014^a^	*MaP* ^*R245W/−*^; *Rosa26*^*LSL−TdTomato/+*^	Metaplastic SC	TNBC	Lung	11
*MaP* ^*−*^ *^/^* ^*−*^					
JM663-T1^a^	*MaP* ^*−*^ *^/^* ^*−*^	Metaplastic SC	TNBC	Lung	12
JM663-T2^a^	*MaP* ^*−*^ *^/^* ^*−*^	Metaplastic SC	TNBC		
JM779-T1	*MaP* ^*−*^ *^/^* ^*−*^	Metaplastic SC	TNBC	No	12
JM779-T2^a^	*MaP* ^*−*^ *^/^* ^*−*^	Nonmetaplastic AC	TNBC		
JM833-T1^a^	*MaP* ^*−*^ *^/^* ^*−*^	Metaplastic PDC	TNBC	Lung	18
JM833-T2^a^	*MaP* ^*−*^ *^/^* ^*−*^	Metaplastic PDC	TNBC		
JM836-T1	*MaP* ^*−*^ *^/^* ^*−*^	Nonmetaplastic AC	TNBC	Lung and Liver	13
JM836-T2^a^	*MaP* ^*−*^ *^/^* ^*−*^	Metaplastic SC	TNBC		

Abbreviations: AC, adenocarcinoma; PDC, poorly differentiated carcinoma; SAC, sarcoma; SC, sarcomatoid carcinoma.

a Tumor was sequenced.

Primary mammary tumors demonstrate diverse histopathology across mice harboring different *p53* missense mutations. Nonmetaplastic and metaplastic histological subtypes were observed. All seven mammary tumors from *MaP*^*R172H/−*^ mice were nonmetaplastic adenocarcinomas (AC; Table 1; Fig. 1C and D). Five *MaP*^*R245W/−*^ mice developed tumors that were both nonmetaplastic (2/5, 20%) and metaplastic (3/5, 80%; Fig. 1C). The nonmetaplastic tumors were a sarcoma (SAC, 1/5, 20%) and adenocarcinoma (AC, 1/5, 20%), whereas the metaplastic tumors were sarcomatoid carcinomas (SC, 3/5, 60%; Table 1; Fig. 1D). Only four of six *MaP*^*−^/^−*^ mice yielded mammary tumors; however, each mouse had two focal mammary tumors for a total of eight tumors of this genotype. These tumors included nonmetaplastic AC (2/8, 25%), metaplastic SC (4/8, 50%), and metaplastic poorly differentiated carcinoma (2/8, 25%; Table1; Fig. 1C and D). Tumors from our somatic mouse model therefore develop both the more common AC histology and rare SAC, SC, and poorly differentiated carcinoma histological subtypes noted in more aggressive human breast cancer cases (55).

To highlight the relevance of the somatic breast model and its ability to mimic human breast cancer molecular subtypes, we characterized mammary tumors associated with each p53 mutant for their expression of *Esr1* (ER), *Pgr* (PR), and *Erbb2* (HER2). The majority of the *MaP*^*R172H/−*^ tumors are of the luminal B subtype, with 86% expressing *Erbb2* and *Esr1*, *Pgr*, or both. The remaining 14% of *MaP*^*R172H/−*^ tumors model HER2-enriched disease, expressing only *Erbb2* (Table 1; Fig. 1E; ref. 25). All *MaP*^*R245W/−*^ mammary tumors were TNBC lacking *Esr1*, *Pgr*, or *Erbb2* expression (Table 1; Fig. 1E)*.* All mammary tumors from *MaP*^*−/−*^ mice were also TNBC (Table 1; Fig. 1E). Collectively, these results demonstrate that our mutant p53 somatic model of mammary cancer develops primary tumors reflecting the human molecular and histological subtypes of breast cancer.

### Somatic breast tumors with p53 mutations are metastatic

**Figure 2:**
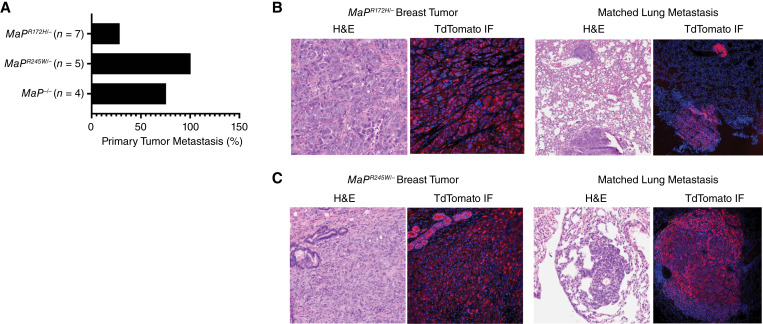
Metastasis in *MaP* mice. **A,** Metastasis incidence in *MaP* mice. **B,** Histology and immunofluorescence staining of TdTomato in a primary breast tumor and matched lung metastasis from a *MaP*^*R172H/−*^ mouse, YZ2. **C,** Histology and immunofluorescence staining of TdTomato in a primary breast tumor and matched lung metastasis from a *MaP*^*R245W/−*^ mouse, JM1233. H&E, hematoxylin and eosin; IF, immunofluorescence.

A metastasis phenotype is associated with *Trp53* mutations (54, 56–63). Distant metastases in our cohorts were determined by gross dissection and histopathology and, in some cases, visualized using TdTomato as a fluorescent reporter (Table 1; Fig. 2). Among the *MaP*^*R172H/−*^ mice, only 28% of the mice (2 out of 7) bearing mammary tumors developed metastasis to the lungs (Table 1; Fig. 2A and B). In the *MaP*^*R245W/−*^ cohort, 100% of the mammary tumors (*n* = 5) were metastatic to the lungs (Table 1; Fig. 2A and C). Immunofluorescence staining for TdTomato revealed recombination in epithelial cells of primary mammary tumors and their lung metastases in both *MaP*^*R172H/−*^ and *MaP*^*R245W/−*^ mice (Fig. 2B and C). In the *MaP*^*−/−*^ cohort, four mice developed primary breast tumors and three of these mice developed metastasis, two with metastasis to the lungs, and one mouse with metastasis to both the lung and liver (Table 1; Fig. 2A).

### Transcriptomes of mutant p53-driven tumors demonstrate mutant-specific pathway enrichment

**Figure 3:**
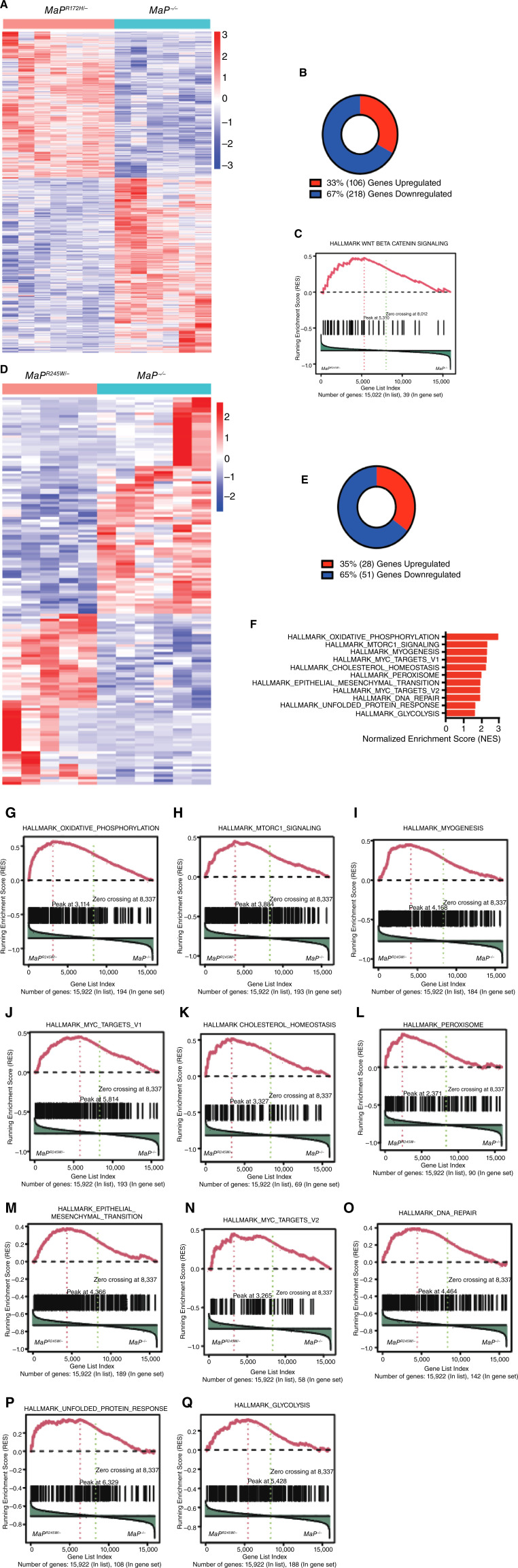
Transcriptomes of *MaP*^*R172H/−*^ and *MaP*^*R245W/−*^ mice disrupt nonoverlapping cancer pathways. **A,** Heatmap showing hierarchical clustering of *MaP*^*R172H/−*^ and *MaP*^*−/−*^ mammary tumors using the Pearson distance and Ward linkage. Order of samples for *MaP*^*R172H/−*^: YZ17, YZ11, YZ4, YZ6, YZ10, YZ2, and YZ3. Order of samples for *MaP*^*−/−*^: JM663-T2, JM779-T2, JM836-T2, JM663-T1, JM833-T1, and JM833-T2. **B,** Pie chart representing significantly DEGs for *MaP*^*R172H/−*^ mammary tumors compared with *MaP*^*−/−*^ mammary tumors (DESeq2, significance criteria were FDR <5% and log_2_ fold change > 2). **C,** Enrichment plot for Wnt Beta Catenin signaling. **D,** Heatmap showing hierarchical clustering of *MaP*^*R245W/−*^ and *MaP*^*−/−*^ mammary tumors. Order of samples for *MaP*^*R245W/−*^: JM1233-T1, JM1126, JM1133, JM1564, and RM0014. Order of samples for *MaP*^*−/−*^: JM836-T2, JM663-T1, JM663-T2, JM779-T2, JM833-T1, and JM833-T2. **E,** Pie chart representing significantly DEGs for *MaP*^*R245W/−*^ mammary tumors compared with *MaP*^*−*/*−*^ mammary tumors. **F,** Representation of significantly enriched Hallmarks pathways as defined by gene set enrichment analysis. **G,** Enrichment plots for pathways involved in oxidative phosphorylation signaling, mTORC1 signaling (**H**), myogenesis signaling (**I**), Myc targets V1 (**J**), cholesterol homeostasis (**K**), peroxisome (**L**), epithelial–mesenchymal transition (**M**), Myc targets V2 (**N**), DNA repair (**O**), unfolded protein response (**P**), and glycolysis (**Q**) in *MaP*^*R245W/−*^ compared with *MaP*^*−l−*^ mammary tumors.

Mutant p53 oncogenic activities have been attributed to its interactions with other transcription factors and disruption of transcriptional networks (27). The transcriptional output associated with these activities becomes evident when comparing tumors with *Trp53* missense mutations to *Trp53* deletion (54). RNA sequencing was performed on *MaP*^*R172H/−*^ (*n* = 7) and *MaP*^*R245W/−*^ (*n* = 5) mammary tumors and compared with *MaP*^*−/−*^ (*n* = 6) mammary tumors (Table 1). Comparison of *MaP*^*R172H/−*^ mammary tumors to *MaP*^*−/−*^ mammary tumors revealed 324 DEGs, of which 106 genes are upregulated and 218 genes are downregulated using an FDR of 5% and a log_2_ fold change of 2 (Fig. 3A and B). To assess pathways dysregulated by p53R172H, GSEA was performed using the Hallmark database of pathway signatures (Fig. 3C; Supplementary Table S2; refs. 46, 64). These analyses show significant enrichment of only the Wnt Beta Catenin signaling pathway (FDR < 0.05; Fig. 3C; ref. 64). A second GSEA was performed using the Reactome database, revealing enrichment for processes involving the formation of the beta catenin TCF-transactivating complex, corroborating the activation of Wnt signaling in *MaP*^*R172H/−*^ mammary tumors (46, 65).

The *TP53*^*R248W*^ missense mutation (human ortholog of mouse *p53*^*R245W*^) is the most prevalent hotspot mutation in breast cancer, associated with poor prognosis and reduced survival when compared with other *TP53* mutations (22, 66, 67). Transcriptome analyses revealed 79 DEGs of which 28 are upregulated and 51 are downregulated when *MaP*^*R245W/−*^ tumors were compared with *MaP*^*−/−*^ tumors (Fig. 3D and E). GSEA revealed significant enrichment of Hallmark signatures associated with oxidative phosphorylation, mTORC1 signaling, myogenesis, MYC Targets V1, cholesterol homeostasis, peroxisome, epithelial–mesenchymal transition, MYC Targets V2, DNA repair, unfolded protein response, and glycolysis (FDR < 0.05; Fig. 3F–Q; Supplementary Table S3; refs. 46, 64). GSEA using the Reactome database revealed enrichment for multiple processes involving electron transport (FDR < 0.05; refs. 46, 65). These data corroborate the Hallmark finding of oxidative phosphorylation as this process involves energy produced from redox reactions of the electron transport chain. We also observed enrichment for cholesterol biosynthesis, a known process regulated by mutant p53 in breast cancer (21).

### *MaP* ^*−/* *−*^ mammary tumors enrich for proinflammatory cytokines and immune signaling

We also examined the cancer-associated pathways that drive *MaP*^−*/*−^ mammary tumors. A reciprocal GSEA was performed to identify pathways enriched in *MaP*^−*/*−^ mammary tumors as compared with *MaP*^*R172H/*−^ and *MaP*^*R245W/*−^ mammary tumors. In comparison with *MaP*^*R172H/*−^ mammary tumors, the gene expression profile of *MaP*^−*/*−^ mammary tumors enriched for Hallmark pathways that included allograft rejection, inflammatory response, MYC Targets V1, complement, protein secretion, and TNFA signaling *via* NFKB (FDR < 0.05; Supplementary Table S4; refs. 46, 64) GSEA utilizing the Reactome database revealed enriched proinflammatory cytokine signaling and other immune signatures, consistent with some of the pathways associated with the Hallmark analysis (FDR < 0.05; refs. 46, 65). GSEA of *MaP*^*−/−*^ mammary tumors compared with *MaP*^*R245W/−*^ tumors demonstrated enrichment for the IFNγ response and IFNα response pathways (FDR < 0.05; Supplementary Table S5; refs. 46, 64). GSEA using the Reactome database revealed that *MaP*^*−/−*^ mammary tumors enrich chemokine signaling (46, 65). In both analyses, cytokine signaling is enriched. Although the Hallmarks and Reactome analyses comparing *MaP*^*−/−*^ mammary tumors to *MaP*^*R172H/−*^ and *MaP*^*R245W/−*^ tumors are distinct, a common theme emerges that proinflammatory cytokines and immune signaling are enriched in *MaP*^*−/−*^ tumors.

### Promoter analysis nominates candidate mediators of p53 mutant-specific transcriptional programs in breast cancer

**Figure 4:**
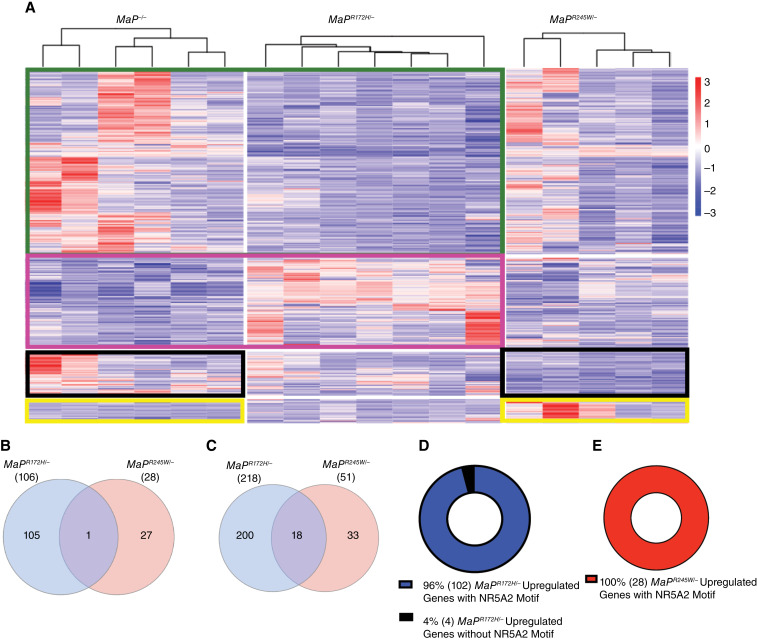
p53R172H and p53R245W show distinct transcriptomes *via* Nr5a2. **A,** Supervised clustering of *MaP*^*−/−*^, *MaP*^*R172H/−*^ and *MaP*^*R245W/−*^ tumors based on upregulated and downregulated genes. Green box, comparison of *MaP*^*R172H/−*^ and *MaP*^*−/−*^ downregulated genes; Pink box, comparison of *MaP*^*R172H/−*^ and *MaP*^*−/−*^ upregulated genes; Black boxes, comparison of *MaP*^*R245W/−*^ and *MaP*^*−/−*^ downregulated genes; Yellow boxes, comparison of *MaP*^*R245W/−*^ and *MaP*^*−/−*^ upregulated genes. **B,** Venn diagrams depicting genes significantly upregulated and (**C**) significantly downregulated in *MaP*^*R172H/−*^ and *MaP*^*R245W/−*^ mammary tumors. **D,** Pie charts representing the number of significantly upregulated genes in *MaP*^*R172H/−*^ tumors and (**E**) *MaP*^*R245W/−*^ tumors that have an Nr5a2 motif.

The transcriptomes of p53R172H- and p53R245W-driven breast tumors demonstrate little overlap. Supervised hierarchical clustering of DEGs from *MaP*^*R172H/−*^, *MaP*^*R245W/−*^, and *MaP*^*−/−*^ mice suggests activation of distinct target genes across the mutants (Fig. 4A). InteractiVenn identified commonly shared DEGs between *MaP*^*R172H/−*^ and *MaP*^*R245W/−*^ mammary tumors (68). Only one upregulated gene and 18 downregulated genes were shared between the DEGs for p53R172H and p53R245W, respectively (Fig. 4B and C).

Motif enrichment analysis of upregulated DEGs for each mutant was performed using MEME-SEA to determine which transcription factor binding motifs were enriched in the 10 kb region upstream of the transcription start sites of all DEGs (bioRxiv 2021.08.23.457422). Genes from analysis of *MaP*^*R172H/−*^ breast tumors show enrichment for 100 transcription factor motifs (FDR < 5.0%; Table 2). Upregulated genes in *MaP*^*R245W/−*^ tumors demonstrate enrichment for 40 transcription factor motifs (FDR < 5.0%; Table 2). To focus on which candidate transcriptional regulators were potentially mediating the transcriptomic changes associated with p53R172H and p53R245W, we identified factors known to activate Wnt signaling and oxidative phosphorylation. Our analysis revealed NR5A2 as a candidate upstream regulator of both Wnt signaling and oxidative phosphorylation (30, 69).

**Table 2:** Transcription factor motifs enriched in *MaP*^*R172H/−*^ and *MaP*^*R245W/−*^ mammary tumors

Transcription factors with enriched motifs in *MaP*^*R172H/−*^ tumors	*q*-value	Transcription factors with enriched motifs in *MaP*^*R245W/−*^ tumors	*q*-value
ZN148	1.85E−27	MAZ	1.08E−09
MAZ	2.75E−24	SP1	1.59E−08
ZBT17	3.86E−24	SP5	1.59E−08
WT1	2.77E−22	SALL1	2.28E−07
SP5	2.77E−22	KLF15	6.24E−07
KLF15	2.22E−19	ZN148	1.41E−06
SP1	3.46E−19	RREB1	1.41E−06
SALL1	1.12E−18	EGR1	1.41E−06
EGR2	7.43E−18	WT1	2.88E−06
RARA	1.24E−17	ARI3A	4.21E−06
RXRA	2.07E−17	EGR2	4.21E−06
ZN281	5.69E−17	ZBT17	4.21E−06
RXRG	1.22E−16	FOXJ3	5.48E−06
SP3	4.87E−16	ZN281	7.15E−06
STF1	2.60E−15	SP1	7.69E−06
KLF6	3.76E−15	EGR2	1.05E−05
KLF3	9.81E−15	SP3	2.04E−05
NR6A1	1.86E−13	ARNT3	2.26E−05
ERR2	3.51E−13	FOXJ3	4.79E−05
FOXJ3	4.43E−13	FLI1	6.07E−05
RREB1	4.43E−13	FOXJ2	6.07E−05
KLF5	5.93E−13	RARA	6.07E−05
FOXJ3	1.90E−12	RXRA	6.93E−05
RXRG	3.15E−12	KLF5	1.76E−04
E2F4	3.35E−12	SP2	2.44E−04
FOXJ2	3.74E−12	ERR2	3.98E−04
SP5	3.74E−12	SP5	3.98E−04
NRF1	1.32E−11	RXRA	4.27E−04
SP4	1.71E−11	EGR4	4.78E−04
RXRA	1.85E−11	HEY2	4.78E−04
SP1	2.30E−11	RXRG	5.05E−04
NR5A2	7.61E−11	NR5A2	5.40E−04
KLF1	2.14E−10	IRF3	6.99E−04
EGR1	2.59E−10	HXC5	8.06E−04
SP2	3.02E−10	KLF15	8.53E−04
E2F1	1.50E−09	PKNX1	8.53E−04
PITX2	3.84E−09	STF1	8.53E−04
ARI3A	1.86E−08	CTCFL	8.53E−04
MAX	1.95E−08	ZN143	8.53E−04
MYC	6.38E−08	KLF5	9.87E−04
THA11	6.67E−08		
MXI1	7.46E−08		
MAX	9.93E−08		
IRF3	1.06E−07		
RXRA	1.95E−07		
NR1I2	4.24E−07		
ZN143	6.07E−07		
FOXF1	7.32E−07		
EGR2	8.74E−07		
MYCN	1.05E−06		
SALL4	1.21E−06		
ELF5	1.82E−06		
STAT2	2.12E−06		
RARG	4.55E−06		
RARA	5.41E−06		
CTCFL	6.27E−06		
E2F7	6.46E−06		
MXI1	6.46E−06		
KLF4	7.03E−06		
KAISO	7.41E−06		
ASCL1	8.62E−06		
ZFX	1.18E−05		
E2F4	1.26E−05		
ETV5	1.67E−05		
HIC1	3.47E−05		
CTCF	4.05E−05		
PURA	4.91E−05		
E2F6	6.31E−05		
BHE41	6.53E−05		
MSX3	9.61E−05		
MITF	1.31E−04		
HXB8	1.44E−04		
FOXI1	1.44E−04		
ZFHX3	1.56E−04		
TAF1	1.74E−04		
RARG	1.83E−04		
ESR2	1.88E−04		
PRDM5	1.88E−04		
PO3F2	1.95E−04		
HXC6	2.01E−04		
AIRE	2.02E−04		
ERR1	2.20E−04		
MAFK	2.22E−04		
NR1H4	2.43E−04		
NFYA	2.57E−04		
NKX28	2.87E−04		
PO5F1	3.10E−04		
SP2	3.27E−04		
NR1I3	4.48E−04		
NFYB	4.92E−04		
JUN	5.15E−04		
NFYC	5.25E−04		
ESR2	5.82E−04		
STAT1	7.30E−04		
HNF4A	7.83E−04		
SRBP2	8.76E−04		
ZBT7A	8.84E−04		
NR0B1	9.20E−04		
FLI1	9.92E−04		

We then used FIMO to determine how many DEGs contained the Nr5a2 motif (53). We found 96% (104/106) of upregulated DEGs in *MaP*^*R172H/−*^ tumors and 100% (28/28) of upregulated DEGs in *MaP*^*R245W/−*^ harbor at least one Nr5a2 binding motif in the promoters (Fig. 4D and E; Supplementary Tables S6 and S7). Our transcriptomic analyses unexpectedly nominate Nr5a2 as a common mediator of the otherwise distinct transcriptional output from p53R172H and p53R245W mutants.

### Human breast tumors harboring p53 mutations recapitulate Nr5a2 as a transcriptional mediator

**Figure 5:**
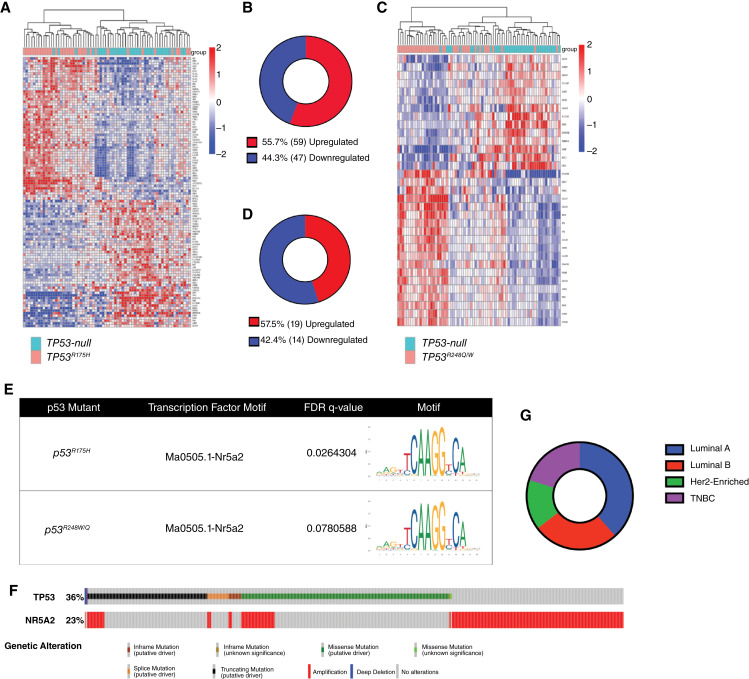
Transcriptomic meta-analysis of human breast tumors with *TP53*^*R175H*^ and *TP53*^*R248W/Q*^ mutations recapitulates NR5A2 as a co-regulator. **A,** Supervised clustering of human breast tumors from METABRIC dataset harboring a *TP53*^*R175H*^ mutation compared with human breast tumors with a homozygous deletion of *TP53*. **B,** Pie chart representing significantly DEGs for *TP53*^*R175H*^ breast tumors compared with *TP53-*null breast tumors (limma), significance criteria were FDR <5% and fold change >1.5. **C,** Supervised clustering of human breast tumors from METABRIC dataset harboring a *TP53*^*R248Q*^ or *TP53*^*R248W*^ mutation compared with human breast tumors with a homozygous deletion of *TP53*. **D,** Pie chart representing DEGs for *TP53*^*R248Q/W*^ breast tumors compared with *TP53*-null breast tumors (limma), significance criteria were FDR <5% and fold change >1.5. **E,** NR5A2 binding motif sequences identified by MEME from JASPAR database. **F,** Oncoprint analysis in cBioportal to assess co-occurrence of *TP53* mutations with *NR5A2* amplifications. **G,** Prevalence of co-occurrence of *TP53* missense mutations with *NR5A2* amplification across human molecular breast cancer subtypes: luminal A (38%), luminal B (26%), HER2-enriched (15%), and TNBC (21%).

To assess if Nr5a2 is a mediator of mutant p53 transcriptional rewiring in human breast cancers, we performed gene expression analyses of the METABRIC consortium, a highly annotated and comprehensive omics analysis of breast cancers (47). We compared human breast tumors harboring either a *TP53*^*R175H*^ or *TP53*^*R248Q/W*^ mutation, the human counterparts to mouse *Trp53*^*R172H*^ and *Trp53*^*R245W*^, respectively, to *TP53*-null tumors. This analysis identified 106 DEGs (59 upregulated and 47 downregulated) in breast tumors harboring a *TP53*^*R175H*^ mutation (Fig. 5A and B). Motif enrichment analysis of upregulated DEGs identified the *NR5A2* motif (FDR = 2.64%) as the most significantly enriched (Fig. 5E; Supplementary Table S8; refs. 51, 52).

A similar analysis was performed for *TP53*^*R248Q/W*^ tumors compared with *TP53*-null breast tumors (Fig. 5C and D). For this analysis, both *TP53*^*R248Q*^ and *TP53*^*R248W*^ breast tumors were combined for a larger sample size. The analysis found 33 DEGs (19 upregulated and 14 downregulated). Motif enrichment performed on regions upstream of the 19 upregulated genes in human *TP53*^*R248Q/W*^ tumors also identified the *NR5A2* binding motif (FDR = 7.81%) as the most significantly enriched result (Fig. 5E; Supplementary Table S8; refs. 51, 52).

To further assess the relevance of *NR5A2* to human breast cancer, we generated an Oncoprint across breast tumors from the METABRIC study (47). This was done to determine whether *NR5A2* is recurrently mutated or amplified in breast cancer and whether *NR5A2* alterations co-occur with *TP53* alterations (48). The METABRIC study is the only publicly available human breast cancer dataset that has mutation and copy number analyses, with 2068 samples representing the four molecular subtypes of breast cancer. Twenty-three percent of these breast cancers harbor an amplification of *NR5A2*; no tumors have *NR5A2* deletions (Fig. 5F). *TP53* is altered in 36% of these tumors, 21% of which harbor a p53 missense mutation. Fifteen percent (65/437) of breast tumors with a *TP53* missense mutation also harbor a concurrent *NR5A2* amplification (Fig. 5F). Each breast tumor with co-occurrence of *TP53* missense mutations and *NR5A2* amplification was annotated for its ER, PR, and HER2 status to examine prevalence across breast cancer subtypes. Our annotations revealed co-occurrence in 38% luminal A, 26%, luminal B, 15% HER2-enriched, and 21% TNBC (Fig. 5G). Thus, this co-occurrence is most prevalent in luminal A breast tumors.

### p53 mutants transcriptionally reprogram Nr5a2 target genes

**Figure 6:**
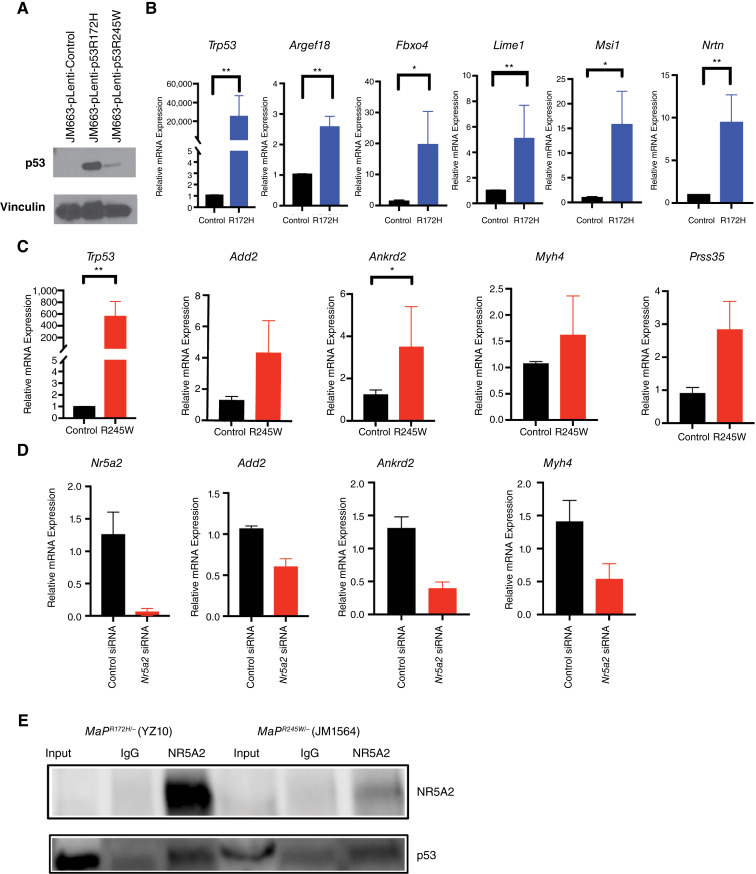
p53R172H and p53R245W interact with Nr5a2 to alter transcription. **A,** Western blot analyses of p53R172H and p53R245W protein levels in *Trp53*-null breast tumor cells infected with lentiviral vectors expressing p53R172H or p53R245W, compared with empty vector control. **B,** qRT-PCR analysis of p53R172H downstream target genes *Argef18, Fbxo2, Lime1, Msi1, Nrtn* with Nr5a2 motifs after lentivirus infection of p53R172H (*n* = 6, *P* value <0.002, 0.01, 0.002, 0.002, 0.03, respectively, Mann–Whitney *U* test). **C,** qRT-PCR analysis of p53R245W downstream target genes *Add2, Ankrd2, Myh4,* and *Prss35* with Nr5a2 motifs after lentivirus infection of p53R245W (*n* = 6, *P* value < 0.002, 0.4, 0.04, 0.6, 0.2, respectively, Mann–Whitney *U* test). C, control. **D,** qRT-PCR analysis of p53R245W downstream target genes *Add2, Ankrd2,* and *Myh4* after siRNA-mediated knockdown of *Nr5a2* in *MaP*^*R245W/−*^ cell line, RM0014. C, control. **E,** Co-immunoprecipitation experiments of *MaP*^*R172H/−*^ cell line YZ10 and *MaP*^*R245W/−*^ cell line JM1564 using Nr5a2 pull-down and probing for mutant p53.

Our *in vivo* and *in silico* transcriptomic analyses predict Nr5a2 to mediate distinct *Trp53*^*R172H*^ and *Trp53*^*R245W*^ transcriptomes. To assess if p53 mutants and Nr5a2 cooperate to alter transcription, we established stable cell lines overexpressing p53R172H or p53R245W in a *p53-null* background, using lentiviral expression vectors. Quantification of mutant p53 protein levels showed a 69-fold increase of p53R172H and a 4.8-fold increase of p53R245W compared with empty vector control (Fig. 6A). Note: Cells overexpressing p53R245W tended to proliferate slower compared with those overexpressing p53R172H, which were highly proliferative. Real-time qPCR also demonstrated statistically significant overexpression of *Trp53*^*R172H*^ (*P* value < 0.002, unpaired *t* test) and *Trp53*^*R245W*^ (*P* value = 0.002, unpaired *t* test) at the RNA level (Fig. 6B and C). Overexpression of *Trp53*^*R172H*^ resulted in significant upregulation of downstream target genes *Argef18*, *Fbxo2*, *Lime1*, *Nrtn1*, and *Msi1* as measured by real-time qPCR (*n* = 6; *P* value < 0.002, 0.01, 0.002, 0.002, 0.03, respectively, Mann–Whitney *U* test; Fig. 5B). Overexpression of *Trp53*^*R245W*^ resulted in upregulation of downstream target genes: *Add2*, *Ankrd2*, *Myh4*, and *Prss35* (*n* = 6, *P* value < 0.4, 0.04, 0.6, 0.2, respectively, Mann–Whitney *U* test; Fig. 6C)*.* The only significantly upregulated gene was *Ankrd2*, whereas all other genes trended upward. Collectively, these results suggest that introduction of the p53 missense mutants into *Trp53*-null mammary tumor cells is sufficient to rewire transcription.

To examine if *Nr5a2* knockdown alters the *Trp53*^*R172H*^ and *Trp53*^*R245W*^ transcriptomes, cell lines from *MaP*^*R172H/−*^ and *MaP*^*R245W/−*^ tumors were transfected with control and *Nr5a2* siRNAs. *MaP*^*R172H/−*^ YZ10 cells died upon *Nr5a2* knockdown, and quantification of targets was not possible. *Nr5a2* knockdown in *MaP*^*R245W/−*^ RM0014 cells resulted in downmodulation of *Nr5a2* and Nr5a2 target genes *Add2*, *Ankrd2*, and *Myh4* (Fig. 6D). These results validate Nr5a2 as the transcriptional mediator of p53 mutant-specific transcriptomes. Therefore, co-immunoprecipitation experiments were performed to determine potential interactions between Nr5a2 and mutant p53. *MaP*^*R172H/−*^ and *MaP*^*R245W/−*^ mammary tumor lysates were immunoprecipitated with an Nr5a2 antibody or IgG. Immunoblotting with a p53 antibody revealed increased mutant p53 levels in the Nr5a2 pull-downs of both the *MaP*^*R172H/−*^ tumor (YZ10), and *MaP*^*R245W/−*^ tumor (JM1564) compared with IgG control, affirming that p53R172H and p53R245W both interact with Nr5a2 (Fig. 6E).

## Discussion

Mutant p53 proteins exert oncogenic activities in many cases through interactions with other transcription factors altering the cellular milieu (27). This study describes a novel association of mutant p53R172H and p53R245W proteins with the transcription factor Nr5a2 in a somatic model of breast cancer. In this model, expression of p53R172H leads to the generation of hormonally driven metastatic breast tumors, whereas expression of p53R245W drives metastatic TNBC. Although the transcriptional programs initiated by the two different p53 proteins were unique for each mutation, both programs were driven *via* Nr5a2. Manipulation of cells in culture (increased expression of mutant p53 and downmodulation of Nr5a2) indicates a direct effect on Nr5a2 transcriptional targets. This interaction represents the gain of function as we purposely generated the model without wild-type p53 to avoid the inhibitory effects of mutant p53 on wild-type p53.

The only transcriptional program associated with p53R172H was the activation of Wnt signaling. Studies have shown that the canonical Wnt signaling pathway mainly controls cell proliferation (70). On the other hand, the p53R245W mutation activates a transcriptional program enriched for multiple pathways associated with aggressive breast cancer including oxidative phosphorylation, mTOR, cholesterol homeostasis, and epithelial–mesenchymal transition. We have previously shown that the p53R245W mutation activates mTOR signaling by enhanced oxidative phosphorylation activity in breast cancer *via* a cooperating mutation in Pip5k1c, and this is corroborated in our transcriptional results (71). Cholesterol biosynthesis has also previously been associated with mutant p53-driven mechanisms in human breast cancer cell lines (21).

Our transcription factor motif analysis yielded many transcription factor motifs enriched upstream of p53R172H and p53R245W specific target genes. Many of these factors were shared across the p53 mutants. We examined each transcription factor on our list to determine its association with Wnt signaling and/or oxidative phosphorylation. Nr5a2 was the only transcriptional factor candidate that has been shown to activate genes involved in both Wnt signaling and oxidative phosphorylation in other contexts (30, 69).

Our meta-analyses of human breast cancer tumors harboring a p53R172H or p53R245W/Q missense mutations corroborate our findings that NR5A2 is a mediator of mutant p53 transcriptional reprogramming. NR5A2 was the top motif identified for both mutants. NR5A2 is an orphan nuclear receptor that regulates embryonic stem cell differentiation and a broad range of functions such as steroidogenesis, cholesterol homeostasis, and tumorigenesis in adult tissues (31). Both NR5A2 and SREBP2 have similar roles in cholesterol homeostasis (72). SREBP2 also binds mutant p53 and disrupts cholesterol biogenesis, leading to disorganized acinar morphology in breast spheroids (21).

Our data indicate that p53R172H and p53R245W lead to transcriptional activation of a nonoverlapping transcriptional program through a single transcription factor Nr5a2. It is possible that p53 mutants engage with Nr5a2 differently to facilitate chromatin accessibility through distinct transcriptional regulators, possibly *via* one or more of the factors in which motifs were also significantly enriched upstream of p53 mutant-specific target genes. Alternatively, NR5A2 has been shown to bind to enhancers and participate in epigenetic modification of chromatin. Thus, the methylation status of the genome may contribute to the differential gene signatures. Further genomic studies such as methylation profiling or ATAC-seq are necessary to determine how the distinct transcriptomes are activated. Lastly, NR5A2 is regulated by ER in luminal breast tumors, demonstrating that hormonal regulation may influence NR5A2 activity (73). The majority of *Trp53*^*R172H/−*^ breast tumors are of luminal origin (Table 1). Thus, it is possible that hormone receptors direct NR5A2 to mediate distinct transcriptional landscapes in breast cancer.

In conclusion, our transcriptome profiling of primary tumors from our somatic model of breast cancer suggests Nr5a2 is a key mediator involved in mutant p53-driven transcriptional rewiring in breast cancers.

## Supplementary Material

Table S1 Sequences for primers used in real-time qRT-PCR studies.

Table S2 Summary of Hallmarks enriched in MaPR172H/- mammary tumors compared to MaP/- mammary tumors.

Table S3 Summary of Hallmarks enriched in MaPR245W/- mammary tumors compared to MaP-/- mammary tumors.

Table S4 Summary of Hallmarks enriched in MaP-/- mammary tumors compared ompared to MaPR172H/- mammary tumors.

Table S5 Summary of Hallmarks enriched in MaP-/- mammary tumors compared ompared to MaPR245W/- mammary tumors.

Table S6 FIMO results summarizing Nr5a2 motif occurrences upstream of p53R172H target gene(s) promoters.

Table S7 FIMO results summarizing Nr5a2 motif occurrences upstream of p53R245W target gene(s) promoters.

Table S8 MEME motif enrichment analysis results summarizing transcription factor motifs significantly enriched in the promoters of significantly upregulated genes of human breast tumors (from METABRIC Consortium) harboring a TP53R175H or TP53R248Q/W mutation.
